# Nucleotide excision repair in Human cell lines lacking both XPC and CSB proteins

**DOI:** 10.1093/nar/gkad334

**Published:** 2023-05-05

**Authors:** Laura A Lindsey-Boltz, Yanyan Yang, Cansu Kose, Nazli Deger, Khagani Eynullazada, Hiroaki Kawara, Aziz Sancar

**Affiliations:** Department of Biochemistry and Biophysics, University of North Carolina School of Medicine, Chapel Hill, NC, USA; Department of Biochemistry and Biophysics, University of North Carolina School of Medicine, Chapel Hill, NC, USA; Department of Biochemistry and Biophysics, University of North Carolina School of Medicine, Chapel Hill, NC, USA; Department of Biochemistry and Biophysics, University of North Carolina School of Medicine, Chapel Hill, NC, USA; Department of Biochemistry and Biophysics, University of North Carolina School of Medicine, Chapel Hill, NC, USA; Department of Biochemistry and Biophysics, University of North Carolina School of Medicine, Chapel Hill, NC, USA; Department of Biochemistry and Biophysics, University of North Carolina School of Medicine, Chapel Hill, NC, USA

## Abstract

Nucleotide excision repair removes UV-induced DNA damage through two distinct sub-pathways, global repair and transcription-coupled repair (TCR). Numerous studies have shown that in human and other mammalian cell lines that the XPC protein is required for repair of DNA damage from nontranscribed DNA via global repair and the CSB protein is required for repair of lesions from transcribed DNA via TCR. Therefore, it is generally assumed that abrogating both sub-pathways with an XPC^−/−^/CSB^−/−^ double mutant would eliminate all nucleotide excision repair. Here we describe the construction of three different XPC^−/−^/CSB^−/−^ human cell lines that, contrary to expectations, perform TCR. The XPC and CSB genes were mutated in cell lines derived from Xeroderma Pigmentosum patients as well as from normal human fibroblasts and repair was analyzed at the whole genome level using the very sensitive XR-seq method. As predicted, XPC^−/−^ cells exhibited only TCR and CSB^−/−^ cells exhibited only global repair. However, the XPC^−/−^/CSB^−/−^ double mutant cell lines, although having greatly reduced repair, exhibited TCR. Mutating the CSA gene to generate a triple mutant XPC^−/−^/CSB^−/−^/CSA^−/−^ cell line eliminated all residual TCR activity. Together, these findings provide new insights into the mechanistic features of mammalian nucleotide excision repair.

## Graphical Abstract

**Graphical Abstract:**
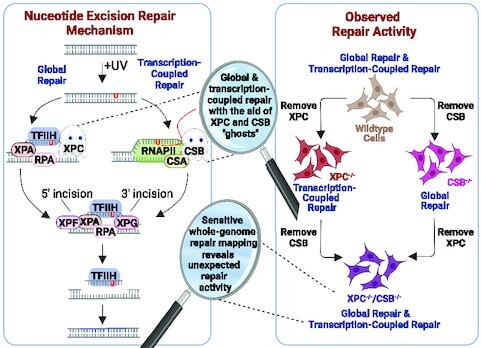
Sensitive whole-genome repair mapping reveals unexpected repair activity in XPC-/-/CSB-/- cells constructed to eliminate both sub pathways of nucleotide excision repair.

## INTRODUCTION

UV damage in the form of cyclobutane pyrimidine dimers (CPDs) and 6–4 pyrimidine-pyrimidone photoproducts [(6–4)PPs] is removed by nucleotide excision repair (excision repair) in humans. The hereditary condition called Xeroderma Pigmentosum (XP) is characterized by increased sensitivity to sunlight and high incidence of skin cancers (1), and genetic analyses have identified seven genes, *XPA* to *XPG*, that are involved in the pathogenesis of this condition due to their role in nucleotide excision repair (2). In addition, two genes, *CSA* and *CSB*, have been shown to participate in a related genetic disorder, Cockayne Syndrome (CS), with photosensitivity resulting from deficient excision repair of UV-induced damage specifically within actively transcribed DNA (3–5). In the years following identification of the XP and CS genes, the corresponding proteins were purified, and nucleotide excision repair was characterized in some detail (6,7). There are two mechanistic pathways: global repair that depends on XPA to XPG and transcription-coupled repair (TCR) that depends on these same factors, with the exception of XPC, and also requires CSA and CSB and several other proteins (8). Global excision repair has been reconstituted with XPA, RPA, XPC, TFIIH, XPF-ERCCI and XPG *in vitro* (9–11), and in this system dual incisions are made to excise the damage in a ∼26-nt-long oligomer. TCR, which acts solely on the transcribed strand independently of the presence or absence of the XPC protein, has not yet been reconstituted *in vitro* and thus the mechanism is less well understood (12,13).

We embarked on this study because our earlier analysis of the repair maps from an XPC-deficient patient-derived cell line (XP-C) revealed that although global repair was greatly diminished, it was not eliminated (14). TCR, which is defined as greater repair of the transcribed strand (TS) than the non-transcribed strand (NTS) of genes actively transcribed by Polymerase II (RNAPII), was clearly observed in the XP-C cell line. Although low in abundance, the presence of excised oligonucleotides mapping to intergenic regions and the NTS of genes did not agree with the universally accepted view that XPC mutants do not perform global repair. Since RNA-seq efforts have led to the view that ‘it appears that almost the entire genome is expressed as RNA’ (15), it is conceivable that the ‘global repair’ seen in the NTS of genes and intergenic regions in XP-C cells was due to TCR from unannotated non-coding or spurious RNAPII transcription. To test this eventuality, we constructed an XPC^−/−^/CSB^−/−^ double mutant cell line with the expectation that this combination would eliminate all repair. We unexpectedly found out that, albeit at greatly diminished levels compared to wild-type counterparts, this newly generated double knockout cell line performs TCR in the absence of CSB and global repair in the absence of XPC.

We proceeded to construct two additional XPC^−/−^/CSB^−/−^ cell lines (Supplementary Figure S1), and tested the repair activity in all the cell lines by UV survival, slot blot analysis of CPD and (6–4)PP removal, and by the *in vivo* excision assay and eXcision Repair-sequencing (XR-seq), which produces a single-nucleotide resolution map of repair genome-wide. In agreement with a previous report (16), we found that XPC^−/−^/CSB^−/−^ cells were extremely sensitive to UV and had essentially undetectable repair activity by the slot blot and *in vivo* excision assays. However, when analyzed for repair with the sensitive XR-seq assay, which directly captures and identifies the excised oligomers to measure repair throughout the genome (14,17), we found that these double mutant cells carry out TCR comparable to XPC mutant cells in terms of the TS/NTS repair ratio, and TCR was completely eliminated in a triple knockout XPC^−/−^/CSB^−/−^/CSA^−/−^ cell line. Quantitative spike-in XR-seq experiments allowed us to determine that nucleotide excision repair in XPC^−/−^/CSB^−/−^ cells was approximately 300-fold less efficient than in wildtype cells and near the limits of detection in the triple knockout cells. Thus, it appears from a mechanistic standpoint that human cells can perform both global repair and TCR in the absence of XPC and CSB. While the physiological relevance of this drastically reduced repair activity remains to be demonstrated, these findings, nevertheless, provide a different perspective to the molecular mechanism of human nucleotide excision repair.

## MATERIALS AND METHODS

### Reagents

Antibodies used in Western analysis were purchased from Santa Cruz (Actin C-11, sc-1615; XPC D-10, sc-74410; CSB E-6, sc-398022) and Abcam (CSA ab137033).

### Biological resources

The normal human skin fibroblast (NHF1) and patient-derived mutant human skin fibroblast cell lines, XP-C (GM15983) and CS-B (GM16095) cell lines were obtained and maintained as previously described (14). Clustered Regularly Interspaced Short Palindromic Repeats (CRISPR)-Cas9 technology was used to generate mutant XPC, CSB, and CSA cell lines (Supplementary Figure S2), and the primers and lentivirus constructs used for the gene-targeting can be found in Supplementary Table S1.

### Survival, slot-blot, excision and XR-seq assays

Survival and slot blot assay procedures have been described previously (18). For all *in vivo* excision and XR-seq experiments, cells were harvested 2h after treatment with 20 J/m^2^ of UVC. XR-seq was performed as previously described (14), except one hundred times more starting material was used for the XPC^−/−^/CSB^−/−^ cell lines which was achieved by combining five batches of twenty 15 cm plates. qXR-seq was conducted by adding 5ul of S2 Hirt lysate [diluted 1:1000 from one 15 cm plate of S2 cells, previously described (19), irradiated with 20 J/m^2^ of UVC and harvested after 1h] to the Hirt lysate from the human cells. All XR-seq and qXR-seq experiments were performed at least two times and representative results are shown.

### Statistical and data analyses

Analysis of sequencing reads and data visualization was performed as described previously (20). Reads were trimmed to remove flanking adapter sequences by cutadapt (21), and then duplicate reads were removed by fastx_toolkit/0.0.14 (hannonlab.cshl.edu/fastx_toolkit/index.html). Trimmed reads were aligned to hg38_UCSC by using bowtie2 with arguments -f -very-sensitive (22). Oligonucleotide lengths and dinucleotide distributions were plotted by R. Only the reads of 24–29-nt in length which contained TT, TC, CC or CT at the expected site of damage were used in the following analysis. For plotting average repair profiles as a unit gene, we chose genes with lengths > 5 kbp, and the distance between genes >5 kb. The genome distribution of XR-seq uniquely mapped reads was determined by CEAS (*cis-*regulatory element annotation system) (23) with command line options ceas -g hg38 -b -w -name.

### Data availability/sequence data resources

The raw data have been deposited in the Sequence Read Archive (SRA) of the National Center for Biotechnology Information (NCBI) under accession number PRJNA933687.

## RESULTS

### DNA repair in XPC^−/−^/CSB^−/−^ cells

Based on numerous studies using assays of various resolution, it has been generally accepted that XPC mutant cell lines carry out TCR but not global repair (24–27). Similarly, it has been reported by several groups that CSB mutant cell lines perform global repair but not TCR (3,4,28). These conclusions, mostly arrived at by using low resolution assays, were more recently confirmed by the XR-seq method (Supplementary Figure S3) that directly measures nucleotide excision repair at a genome-wide scale and at single nucleotide resolution (14). Despite this background, we wanted to find out whether a double mutant of XPC^−/−^/CSB^−/−^ would be completely defective in excision repair as extrapolated from the properties of the individual mutants. Previously, an *xpc*^−/−^/*csb*^−/−^ mouse strain was constructed and fibroblasts from this strain were tested for UV sensitivity (16). This double mutant was much more sensitive than the individual mutants as predicted. However, the repair of UV photoproducts was not measured in that study and therefore the presence of a low level of residual XPC and CSB-independent repair activity remained a possibility.

**Figure 1.**
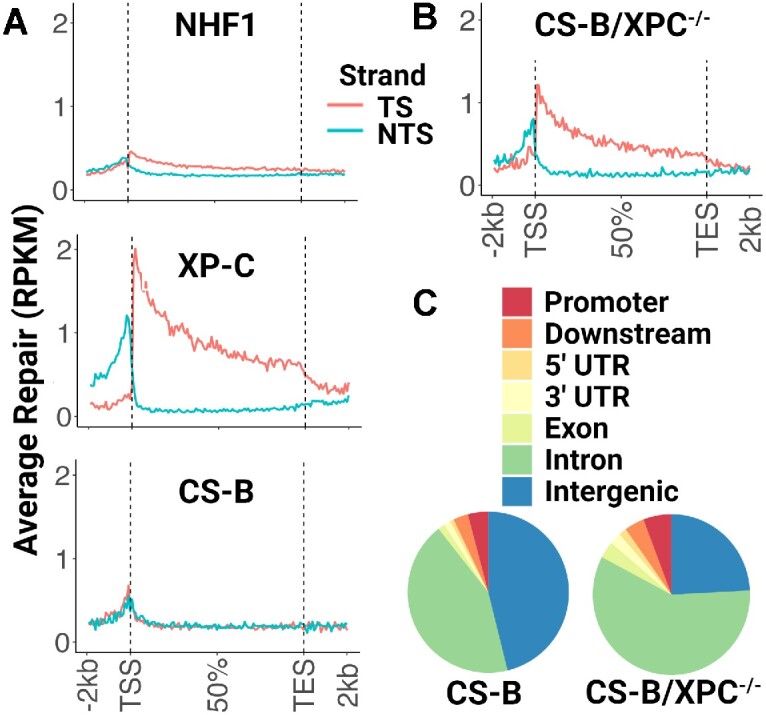
Transcription-coupled repair in patient-derived CS-B/XPC^−/−^ cells. (**A, B**) CPD XR-seq data from the indicated cell lines is plotted as average repair reads (y-axis) along the length of a ‘unit gene’ (x-axis) (divided into 100 bins; 10100 genes were selected for length >5 kb and no overlaps with a distance of at least 5 kb between genes). (**A**) As predicted for repair of CPDs in humans, normal human fibroblasts (NHF1) exhibit only a small amount of TCR due to predominant global repair, patient-derived XP-C cells which lack the XPC protein required for global repair exhibit only TCR, and patient-derived CS-B cells which lack the CSB protein required for TCR exhibit only global repair. (**B**) Unexpectedly, when XPC is mutated in the patient-derived CS-B cells, transcription-coupled repair is now detectable. RPKM, reads per kilobase per million mapped reads; TSS, transcription start site; TES, transcription end site. TS, transcribed strand; NTS, nontranscribed strand. (**C**) Repair as a function of genomic location. The parental patient-derived CS-B cell line, which exhibits only global repair, has the largest proportion of repair events in intergenic regions, whereas in the CS-B/XPC^−/−^ cells most of the repair reads map to genes, consistent with repair by TCR.

We decided to investigate whether repair activity could be detected in the double mutant XPC^−/−^/CSB^−/−^ cells using the very sensitive XR-seq assay to address this eventuality. Figure 1A shows genome-wide repair of UV-induced CPDs in the TS and NTS of wild-type (WT) normal human fibroblasts (NHF1) and XP-C and CS-B patient cells as determined by XR-seq. As can be seen from the figure, and in agreement with previous results (14), there is TCR in WT which is much more pronounced in the XP-C cell line that is lacking global repair; and the CS-B patient cell line showed no TCR. Thus, we decided to knock-out XPC in the CS-B patient cell line (Supplementary Figure S2A) to generate CS-B/XPC^−/−^ cells with the expectation that these cells would be totally repair-defective. Supplementary Figure S4 (lane 4) shows the lack of both XPC and CSB proteins in this cell line, yet analysis of repair by XR-seq (Figure 1B) revealed that in the double mutant, TCR, as measured by the TS/NTS repair ratio, was restored to levels closer to what is seen in the XP-C mutant cell line. Figure 1C shows an analysis of the genome distribution of all repair events. In the parental CS-B cell line, which exhibits only global repair, the largest proportion of repair events are in intergenic regions of the genome, whereas in the CS-B/XPC^−/−^ cells the majority of the repair reads map to genes which is consistent with repair occurring primarily by TCR in this cell line.

Because of this unexpected finding of TCR in cells lacking CSB, we decided to perform the reciprocal knockout of CSB in the XP-C patient cell line to generate XP-C/CSB^−/−^ cells (Supplementary Figure S2B). Supplementary Figure S4 (lane 8) shows the lack of both XPC and CSB proteins in this cell line, and yet, as with the CS-B/XPC^−/−^ double mutant, XR-seq analysis revealed the presence of TCR in the XP-C/CSB^−/−^ cells (Supplementary Figure S5A).

**Figure 2.**
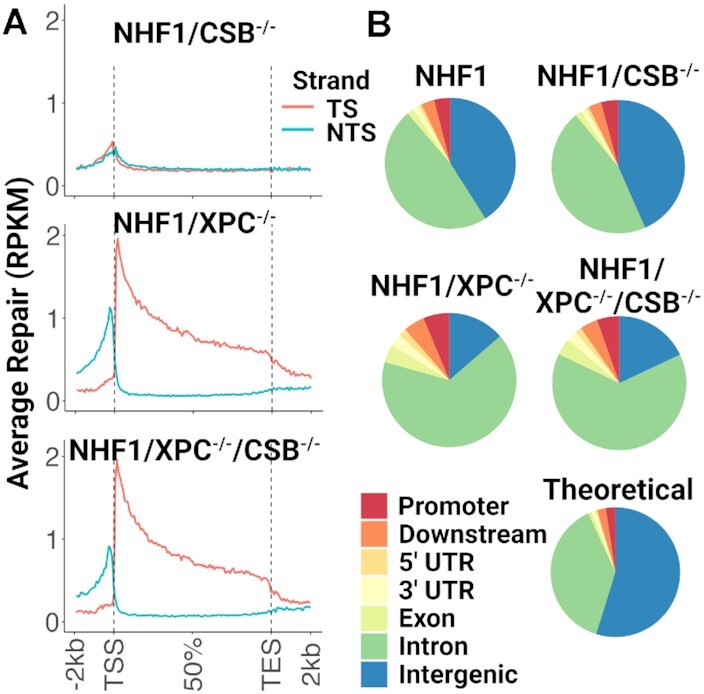
Transcription-coupled repair in normal human fibroblast double knockout NHF1/XPC^−/−^/CSB^−/−^ cells. (**A**) CPD XR-seq data from the indicated NHF1 cell lines is plotted as in Figures1A, B and shows a similar CPD repair pattern in normal human fibroblasts as was seen in the patient-derived cells. (**B**) CPD repair as a function of genomic location in NHF1 cells was analyzed as in Figure 1C.

Since both double mutant CSB^−/−^/XPC^−/−^ cell lines described here are patient-derived and may have unstable genomes due to SV40 immortalization, we considered the possibility that there could be low levels of XPC or CSB protein due to exon skipping, translational frameshifting, or similar rare events. To address this potential problem, we decided to knockout both XPC (Supplementary Figure S2C) and CSB (Supplementary Figure S2D, E) proteins in the NHF1 background. In Supplementary Figure S4, Western blot analysis shows that these proteins are not detectable in the NHF1 cell lines in which CSB, XPC and XPC/CSB were mutated, (lanes 2, 5, and 6, respectively). When these mutants were analyzed by XR-seq for CPD excision repair (Figure 2A), data quite similar to the other two CSB^−/−^/XPC^−/−^ patient-derived cell lines were observed: no genome-wide TCR observed in NHF1/CSB^−/−^ cells but significant TCR is seen in both NHF1/XPC^−/−^ and NHF1/XPC^−/−^/CSB^−/−^ cells. Interestingly, when the genomic location of the repair events for all four cell lines was analyzed (Figure 2B), NHF1/XPC^−/−^/CSB^−/−^ cells had a higher percentage of reads in intergenic regions than the NHF1/XPC^−/−^ cells (the averages from two experiments is 27% and 14%, respectively) indicating that there is global repair in the double knockouts. This observation can also be seen when comparing the genomic location of repair events between the XP-C and XP-C/ CSB^−/−^ patient-derived cell lines (Supplementary Figure S5B).

### Properties of the excision products in the repair of mutant cell lines

**Figure 3.**
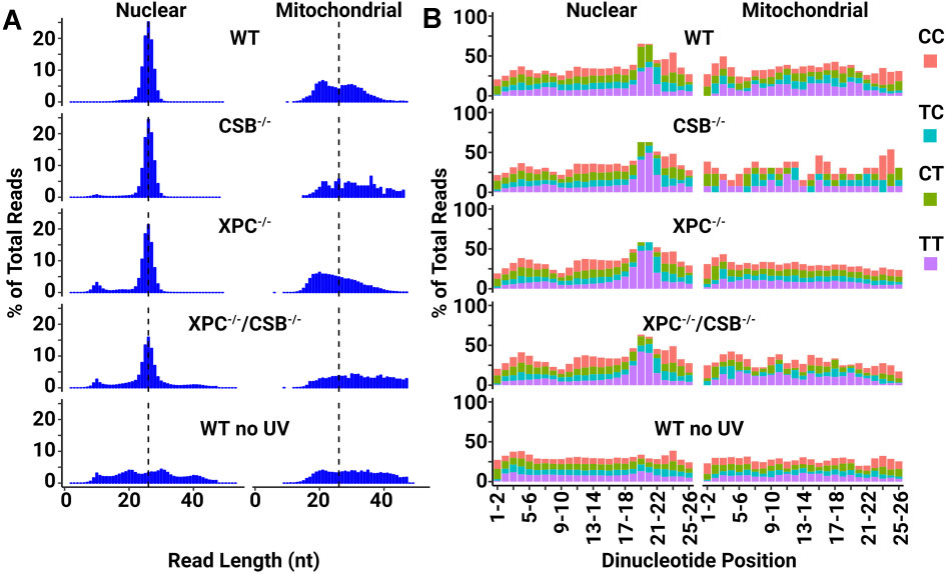
NHF1/XPC^−/−^/CSB^−/−^ cells excise UV photoproducts by the same dual incision mechanism as wild type cells. (**A**) Length distribution of XR-seq reads from the indicated NHF1 cell lines mapped to either nuclear DNA (left) or mitochondrial DNA (right). The 26-nt median is indicated with a dashed line. (**B**) Analysis of the frequency of each of possible dipyrimidine along XR-seq reads of 26-nt length mapped as in (A). Mitochondrial DNA analysis and unirradiated cells (WT no UV, performed on the same 100× scale as the double knockout cells) were included to control for specificity.

In normal human cells, nucleotide excision repair removes UV-induced CPDs and (6–4)PPs in the form of 26–29-nt oligomers by dual incisions 19–21 nt to the 5′ and 5–6 nt to the 3′ of photoproducts (14,29). To find out whether the excision products in the XPC^−/−^/CSB^−/−^ cell lines were produced by the same dual incision mechanism, we analyzed the length distribution (Figure 3A, Supplementary Figure S6A) and sequence composition (Figure 3B, Supplementary Figure S6B) of the XR-seq reads that are mapped to either nuclear or mitochondrial genomes. The mitochondrial DNA fragments (right) and random genomic DNA fragments from unirradiated cells (WT no UV), which are presumable nonspecifically immunoprecipitated (IP) during the XR-seq procedure, do not exhibit the excision repair size distribution or base distribution seen in the excised oligos that map to the nuclear genome (left). The size distribution of excised oligomers in the range of 26–29-nt oligomers and sequence composition of Pyr-Pyr 19–21 nt from the 5′ and 5–6 nt from the 3′ termini show similar patterns in the WT and mutant cell lines. Taken together our data lead to the conclusion that the XPC^−/−^/CSB^−/−^ cells excise UV photoproducts by the same dual incision mechanism as wild type cells.

### UV sensitivity and repair in the mutant cell lines

**Figure 4.**
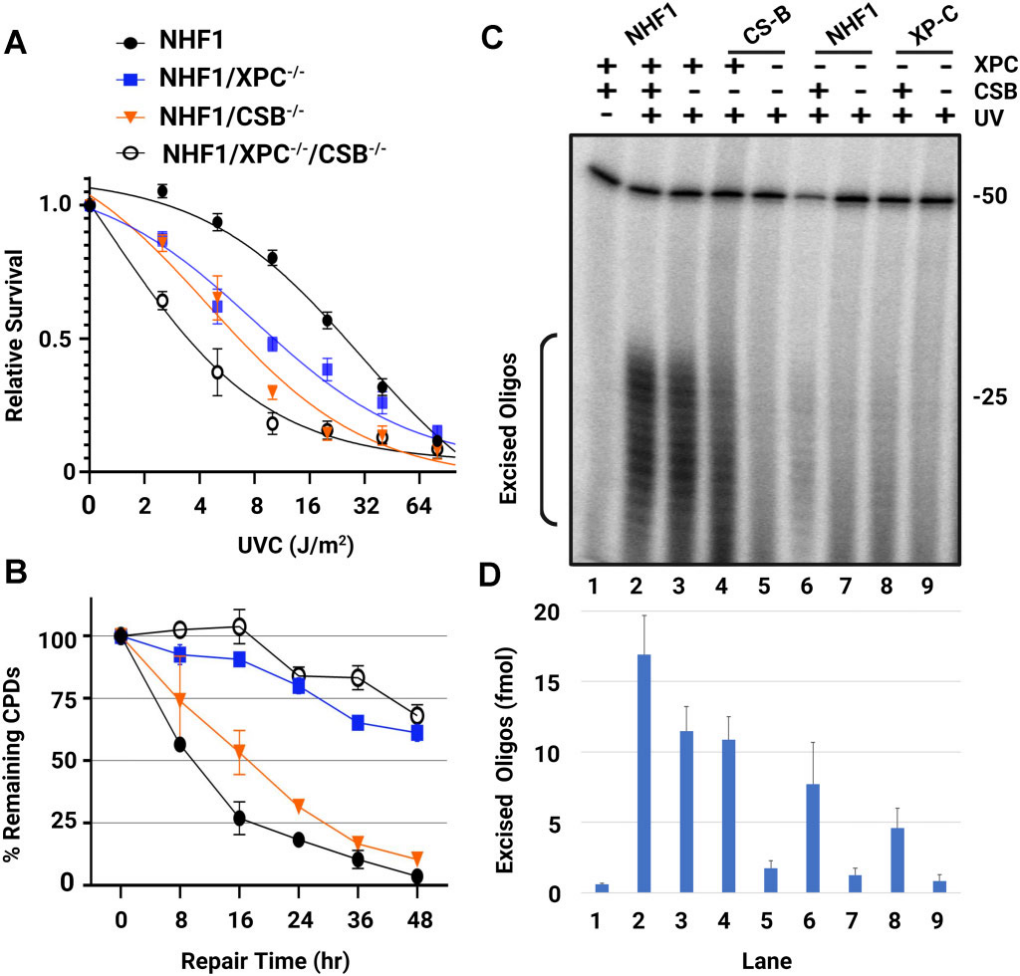
Extreme UV sensitivity and nearly undetectable repair in the XPC^−/−^/CSB^−/−^ cell lines (**A**) The four NHF1 cell lines were analyzed for survival two days after the indicated doses of UVC. Shown are the mean from three biological replicates with error bars denoting standard deviation (SD). (**B**) Slot blot analysis showing CPD repair rates of the four NHF1 cell lines treated with 5 J/m^2^ UVC. CPD signals were normalized to time = 0 and plotted as a function of time. Results shown are the mean from three biological replicates with error bars denoting SD. (**C**) The *in vivo* excision assay was used to compare the amount of CPD-containing excised oligos in extracts from three the parental and XPC^−/−^/CSB^−/−^ cell lines (double knockouts in lanes 5, 7, 9 with the parental strain indicated above). An equal number of cells were irradiated with 20 J/m^2^ UVC and incubated 2 h at 37°C to allow for repair. Cells were lysed by the Hirt procedure and low molecular weight DNA in the supernatant was immunoprecipitated with anti-CPD antibodies. The recovered oligos were mixed with a 50-mer internal control oligo, 3′-end labeled, and separated on a DNA sequencing gel along with the indicated size markers. (**D**) Quantification of three biological replicates of the excision assays above showing the mean with error bars denoting SD.

In Figures 4A and Supplementary Figure S7 we analyzed the survival of the cell lines after exposure to different UV doses and found that the double mutant lines are more sensitive to UV than the single mutants, as was observed with the mouse csb^−/−^/xpc^−/−^ fibroblasts (16). To compare the rate of UV-adduct removal in the four NHF1 cell lines, the slot blot method with damage-specific antibodies was used to measure the dynamic loss of total genomic DNA damage. The cells were irradiated with 5 J/m^2^ of UVC, and as seen in Figure 4B, we observed that about half of the CPDs are removed within 8h in WT NHF1 and within 16h in the NHF1/CSB^−/−^ cells. In contrast, both NHF1/XPC^−/−^ and NHF1/XPC^−/−^/CSB^−/−^ cells required longer than 48h for half of the CPDs to be repaired, with the caveat that measurements at late timepoints are confounded by dilution because of cell division or cell death. Removal of (6–4)PPs is much faster, with essentially all being removed by 1 h in both WT NHF1 and NHF1/CSB^−/−^ cell lines (Supplementary Figure S8A). Only half is removed within 16 h in NHF1/XPC^−/−^ cells, and the rate is even slower in the double mutant cells. Together, these results indicate that very little repair is occurring in XPC^−/−^/CSB^−/−^ cells.

Another way to directly compare nucleotide excision repair between cell lines is to radiolabel the excised oligos purified from an equivalent number of cells (*in vivo* excision assay). As seen from the gel in Figure 4C and from the quantitation in Figure 4D, all three cell lines lacking both XPC and CSB (lanes 5, 7, 9) had levels of excised CPD-containing oligos close to the background signal seen in the unirradiated control (lane 1). A similar result was observed when (6–4)PP-containing oligos were analyzed (Supplementary Figure S8B, C). We performed titration experiments (Supplementary Figure S9) and determined that diluting WT NHF1 ∼100-fold gives a signal equivalent to the double knock out cell lines, which also correlates with the 100-fold more double knockout cells required to perform XR-seq than for WT cells.

### Quantitative XR-seq method

Since nucleotide excision repair in the CSB^−/−^/XPC^−/−^ cell lines was nearly undetectable by standard assays (slot blot and excision assays) but was detectable by XR-seq, we decided to enhance the XR-seq protocol to make it quantitative (qXR-seq, Supplementary Figure S10A) in order to measure the relative differences in repair between WT and CSB^−/−^/XPC^−/−^ cells. To this end, we spiked in an equal quantity of Hirt extract DNA from *Drosophila* S2 cells (that had been UV-irradiated and allowed to repair for 1h) into the extracts from UV-irradiated human cells before performing the IP steps of XR-seq. Determining the ratio of human to *Drosophila* excision products allows for direct comparison between samples since the *Drosophila* extract is constant in all samples. We had to switch from using anti-TFIIH antibodies that were used in the previous experiments to using anti-damage-specific antibodies for the IPs to capture both human and fly excised oligos since the TFIIH antibodies do not recognize the *Drosophila* protein. This switch also allowed us to use the denaturing Hirt extraction procedure which is much more efficient than the nondenaturing lysis procedure required for the TFIIH IPs, thus having the added benefit of requiring much less starting material. In addition, we also discovered that the XR-seq method is generally more efficient for (6–4)PPs than CPDs, which allowed for even less starting material, so with all these changes we were able to use 100-fold less starting material for qXR-seq than we had used in the previously described experiments. Supplementary Figure S10B shows the results from a serial dilution of NHF1 using the qXR-seq method. Since the mapped human/fly oligo ratio relative to cell number is essentially linear, the amount of excised oligos from different cell lines can be determined relative to NHF1, and thus, we were able to determine that nucleotide excision repair in XPC^−/−^/CSB^−/−^ cells is approximately 0.3% of wildtype NFH1 cells.

**Figure 5.**
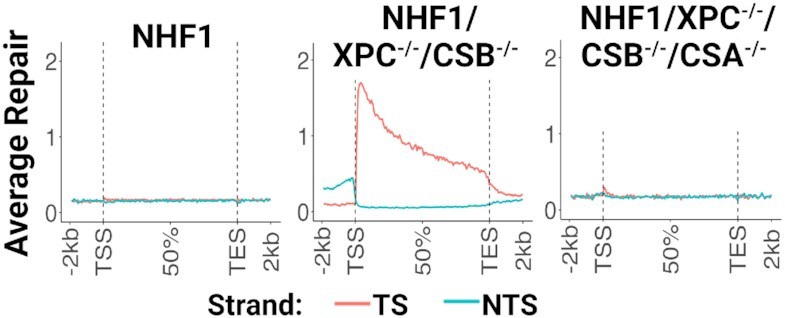
Mutating the CSA gene to generate a triple mutant NHF1/XPC^−/−^/CSB^−/−^/CSA^−/−^ cell line eliminates TCR activity. qXR-seq data plotted as in Figures 1 and 2 show that UV-induced (6–4)PP damage is repaired by TCR in XPC^−/−^/CSB^−/−^ cells (middle), but not in WT NHF1 (left) or XPC^−/−^/CSB^−/−^/CSA^−/−^ cells (right).

Figure 5 shows the qXR-seq results for repair of UV-induced (6–4)PPs in WT (left) and double knockout XPC^−/−^/CSB^−/−^ cells (middle). The lack of obvious (6–4)PP repair by TCR in WT NHF1 cells was as expected, since (6–4)PPs are very efficiently removed by global repair (14). In contrast, NHF1/XPC^−/−^/CSB^−/−^ cells excised (6–4)PP by TCR, similar to what was observed for CPDs (Figure 2A). Since nucleotide excision repair in the double knockout cells was unexpected, as it is XPC- and CSB-independent, yet exhibits TCR, contrary to consensus view of mammalian excision repair in general and the requirement for CSB for TCR in particular, we decided to knock out another component of the TCR pathway to assess whether the observed TCR activity could occur via a novel mechanism. The generally established human TCR mechanism (30,31) consists of damage-stalled RNAPII recruiting CSB translocase and CSA complexed with CRL4 (cullin-ring type E3 ubiquitin ligase 4). This complex then recruits the UVSSA scaffold and ELOF1 which act in concert to ubiquitylate residue K1268 on the largest subunit of RNAPII to ultimately recruit TFIIH and then the other excision repair factors. Thus, we decided to knock out CSA from the NHF1/XPC^−/−^/CSB^−/−^ cells to generate a triple mutant cell line (Supplementary Figure S2F, S4B). We performed qXR-seq on these cells and found that the level of repair was near the bottom limit of the quantitative assay at approximately 0.05% of wildtype NHF1 cells, and that more importantly, the NHF1/XPC^−/−^/CSB^−/−^/CSA^−/−^ cells were totally defective in TCR (Figure 5, right). Taken together, these results lead us to conclude that, like *Drosophila* (19), humans can perform TCR in the absence of CSB, albeit it is very inefficient in human cells. For physiologically relevant levels of TCR, human cells require the CSA and CSB proteins.

## DISCUSSION

**Figure 6.**
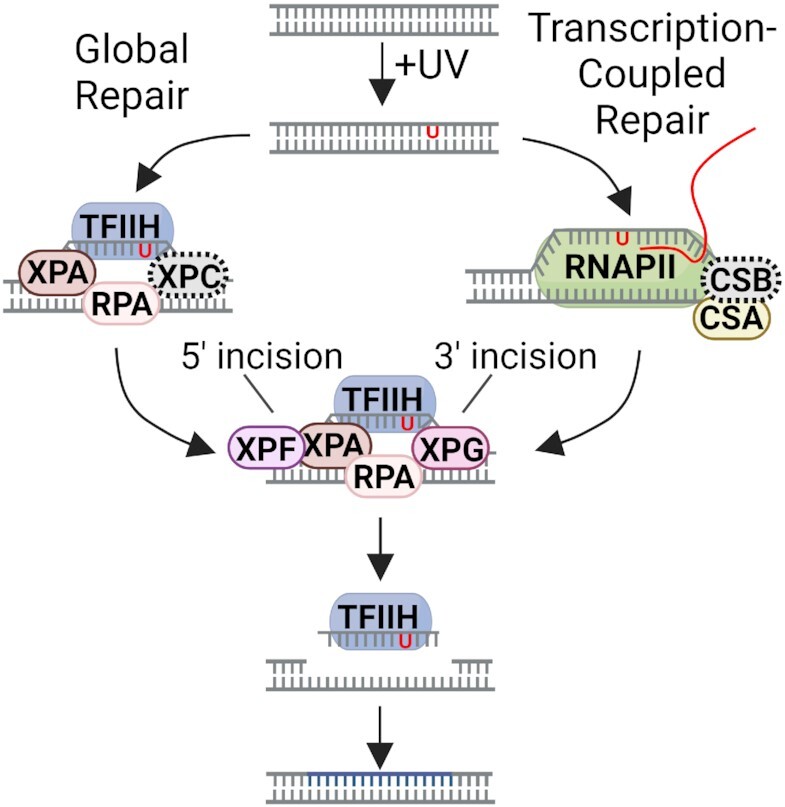
Model for global and transcription-coupled repair (TCR) of UV-induced DNA damage in human cells lacking XPC and CSB proteins. In global repair (left), in the absence of XPC, which is represented as a gray dashed oval, the dual incision complex is assembled at a damage site by binding of XPA-RPA to damage and recruitment of TFIIH by its interaction with XPA. Recruitment of the XPG and XPF nucleases enables dual incisions generating a 26–27-nt long oligomer. In TCR (right), in the absence of CSB, represented as a gray dashed oval, the transcription bubble replaces the XPC damage recognition-function and enables the assembly of the 5 excision factors (XPA, RPA, TFIIH, XPG, and XPF). Excision occurs in the absence of XPC and CSB, but it is drastically reduced because assembly of the 5-factor nuclease is inefficient without these two proteins. Adapted with permission from (43).

In light of the findings reported in this study, we propose the following model for global and transcription-coupled repair in human cells lacking XPC and CSB (Figure 6). In the standard model for global repair, the dual incision complex is assembled at the damage site by binding of XPA, XPC and RPA to damage and recruitment of TFIIH which results in unwinding of the duplex around the damage. This is then accompanied by recruitment of the XPG and XPF nucleases to enable the classic dual incision pattern generating 26–27-nt long oligomers. In the standard model of transcription-coupled repair, the dual incision complex is assembled at the damage site after being encountered by RNAPII which recruits CSB and CSA, and thus initiates a series of events ultimately resulting in the recruitment of TFIIH and the other excision repair factors except XPC.

Our findings at face value seem contradictory to the consensus models for 6-factor (XPA, RPA, XPC, THIIH, XPG, XPF-ERCC1)-dependent global repair and CSB-dependent transcription-coupled repair in humans. However, taking into account the drastically reduced global repair with just five of the six canonical excision repair factors and of TCR in the absence of both CSB and XPC, our data can be reconciled with the standard models when prior findings on excision protein-DNA and protein-protein interactions are taken into account. It has been previously reported that RPA and XPA act cooperatively to recognize DNA damage (32) and that XPA interacts with TFIIH (33). The two nucleases are recruited by interactions of XPG with RPA (32) and TFIIH (9) and XPF-ERCC1 with XPA (34). Thus through these interactions an XPC-independent dual incision complex assembles at a damage site, similar to the Preincision Complex 3 (PIC3) which assembles with the aid of XPC but does not contain XPC (35), and carries out the excision reaction. This is consistent with the random assembly and kinetic proofreading model for damage recognition (36,37). However, this assembly and excision is not as efficient as the excision complex that forms with the aid of the scaffold XPC protein and consequently, the repair rate is drastically reduced.

With respect to TCR in the absence of CSB, we note two previous *in vitro* studies: one which showed that RNAPII stalled at a dimer does not inhibit excision repair nor is CSB required (38), and a second which showed that a dimer in a 10-nt pseudo transcription bubble (no RNAPII but a CPD at the 5′ end of 10 mispaired base pairs) was excised by the conventional dual incision mode (26–27-mer) by 5 repair factors (XPC omitted) (39). These studies indicate that repair could occur at RNAPII-stalled dimers and that the ‘transcription bubble’ could replace the XPC-damage recognition function and enable the assembly of the 5 excision repair factors (XPA, RPA, TFIIH, XPG, XPF). Thus, it is reasonable to assume that similar reactions could occur at low frequency and manifest as TCR in the absence of XPC and CSB. However, our observation that the elimination of both CSB and CSA proteins abrogates all residual TCR indicates that even though a low level of TCR occurs in the absence of CSB, removal of both proteins eliminates the ability of the cell to perform TCR and thus, the TCR mechanism appears to be more complex than currently appreciated.

A plausible explanation for the observation of TCR in the XPC^−/−^/CSB^−/−^ cell lines but not in the CSB^−/−^ cell lines is that in the latter there is still substantial global repair mediated by the 6-factor excision nuclease that obscures the drastically reduced TCR in the CSB^−/−^ cell lines, whereas in the XPC^−/−^/CSB^−/−^ cell lines the ‘global repair’ is drastically reduced making it possible to observe TCR in the background of very low global repair. We did not observe obvious differences in the pattern of TCR between WT cells and those lacking CSB, which is different than what was observed in *Saccharomyces cerevisiae*, which exhibit residual TCR at transcription start sites in the absence of Rad26/CSB (40). Of note, yeast have CSB and CSA orthologs (41), but *Drosophila* do not, yet still perform robust TCR (19,42). Yeast and flies are also unique in that they are the only eukaryotes known to require XPC for TCR (19,41). Taken together, these findings provide a unique perspective to the molecular mechanisms of global and transcription-coupled nucleotide excision repair.

## DATA AVAILABILITY

The raw data have been deposited in the Sequence Read Archive (SRA) of the National Center for Biotechnology Information (NCBI) under accession number PRJNA933687.

## Supplementary Material

gkad334_Supplemental_File
