# Supplementary material for: Nucleotide excision repair in Human cell lines lacking both XPC and CSB proteins
Source: Nucleic Acids Res. 2023 May 5;51(12):6238–45. doi: 10.1093/nar/gkad334 (PMC10325923; doi:10.1093/nar/gkad334)
Supplement: gkad334_Supplemental_File [file gkad334_supplemental_file.docx]

**SUPPLEMENTAL MATERIAL**

Nucleotide Excision Repair in Human Cell Lines Lacking Both XPC and CSB Proteins

**Laura A. Lindsey-Boltz^1^, Yanyan Yang^1^, Cansu Kose, Nazli Deger, Khagani Eynullazada, Hiroaki Kawara, Aziz Sancar^*^**

| Name | Primer Sequence (5’-3’) | Description |
| --- | --- | --- |
| XPC sgRNA#1F | CACCGTAGTAGGTGTCCACATCTCG | For deleting XPC from CS-B to make CS-B/XPC^-/-^ and XPC from NHF1 to make NHF1/XPC^-/-^. LentiCRISPRv2 Blast plasmid (Addgene 83480) was used to target TAGTAGGTGTCCACATCTCG (PAM sequence AGG) with Blasticidin selection. |
| XPC sgRNA#1R | AAACCGAGATGTGGACACCTACTAC |  |
| CSB sgRNA#1F | CACCGCGTGGAGAAGGAGTATCGGT | For deleting CSB from NHF1 to make NHF1/CSB^-/-^ and CSB from NHF1/XPC^-/-^ to make NHF1/XPC^-/-^/CSB^-/-^. LentiCRISPRv2 Hygro plasmid (Addgene 98291) was used to target CGTGGAGAAGGAGTATCGGT (PAM sequence CGG) with Hygromycin selection. |
| CSB sgRNA#1R | AAACACCGATACTCCTTCTCCACGC |  |
| CSB sgRNA#2F | CACCGGTCTGAGTATTTCCCCACAG | For deleting CSB from XP-C to make XP-C/CSB^-/-^. LentiCRISPRv2 Hygro plasmid (Addgene 98291) was used to target GTCTGAGTATTTCCCCACAG (PAM sequence AGG) with Hygromycin selection. |
| CSB sgRNA#2R | AAACCTGTGGGGAAATACTCAGACC |  |
| CSA sgRNA#1F | CACCGCAACTTTGTGACTTGAAGTC | For deleting CSA from NHF1/XPC^-/-^/CSB^-/-^ to make NHF1/XPC^-/-^/CSB^-/-^/CSA^-/-^. LentiCRISPRv2 Neo plasmid (Addgene 98292) was used to target CAACTTTGTGACTTGAAGTC (PAM sequence TGG) with G418 selection. |
| CSA sgRNA#1R | AAACGACTTCAAGTCACAAAGTTGC |  |

**Supplemental_Table_1** The primers, lentivirus constructs, and the antibiotic selection used for CRISPR-Cas9 gene-editing. Lentivirus plasmids expressing the indicated gRNA targets were designed using CHOPCHOP. These were used together with the envelope plasmid pCMV-VSV-G (Addgene 8454) and packaging plasmid psPAX2 (Addgene 12260). The indicated antibiotics were added to cell cultures diluted such that resistant colonies could be isolated from a single originating cell. Isolated single clones were screened by immunoblot and the gene mutations were identified by Sanger sequencing as shown in Fig S2.

**
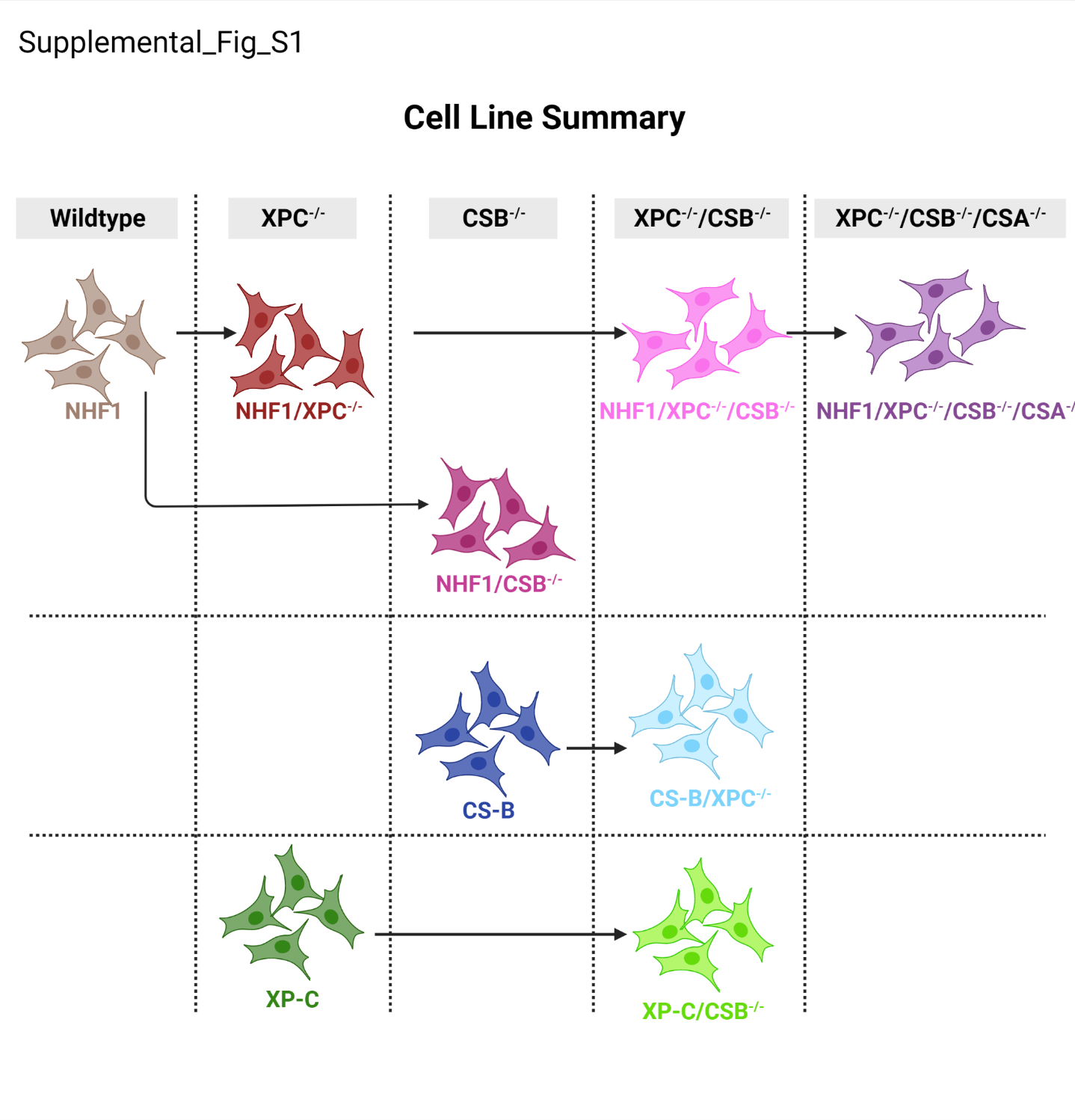
**

**Figure S1. Summary of the cell lines used in this study.** This study analyzed two different XPC^-/-^ cell lines (patient-derived XP-C and normal human fibroblast NHF1/XPC^-/-^), two different CSB^-/-^ cell lines (patient-derived CS-B and NHF1/CSB^-/-^), three different double knock out XPC^-/-^/CSB^-/-^ cell lines, and one triple knock out NHF1/XPC^-/-^/CSB^-/-^/CSA^-/-^ cell line.

**
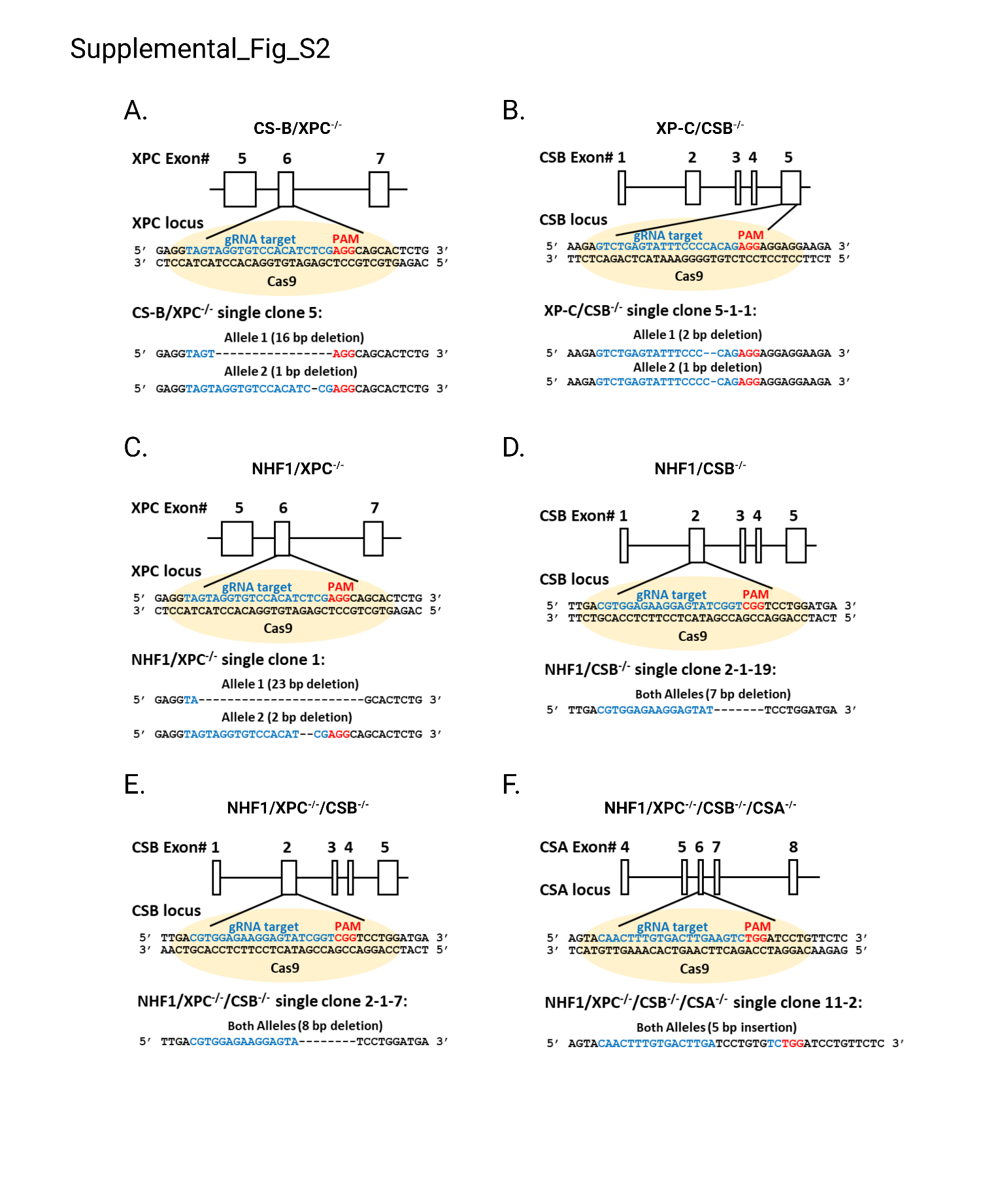
**

**Figure S2.** **Clustered Regularly Interspaced Short Palindromic Repeats (CRISPR)-Cas9 Technology used to generate mutant cell lines.** The sequences and exon locations of the targeted loci are indicated: **(A,C)** XPC; **(B,D,E)** CSB; and **(F)** CSA. Lentivirus plasmids expressing gRNA targets (indicated in blue, are listed in Supplemental_Table_1). Single clones were isolated that lacked the indicated proteins as determined by immunoblot (Fig. S4), and the gene mutations were determined by Sanger sequencing and are indicated at the bottom of each panel.

**
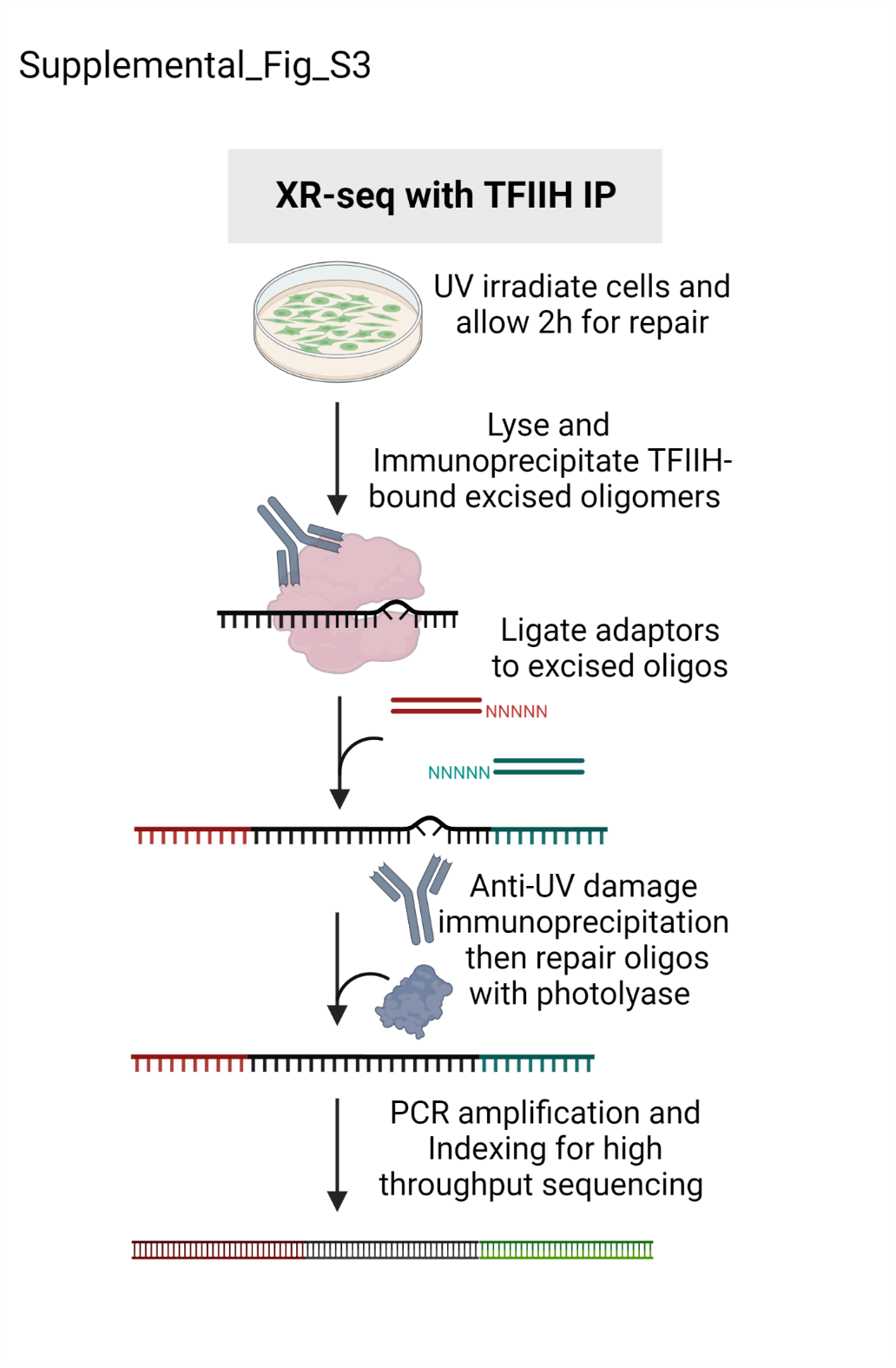
**

**Figure S3. The Excision Repair-sequencing (XR-seq) Method.** This method was used in Figures 1-3 to directly map the genomic location of excision products, which were isolated from lysed cells, purified with anti-TFIIH antibodies, ligated to adapters, and then purified with anti-CPD specific antibodies. The damage was then reversed with CPD photolyase and then PCR was performed to generate libraries for high throughput sequencing. Adapted with permission from (30).

**
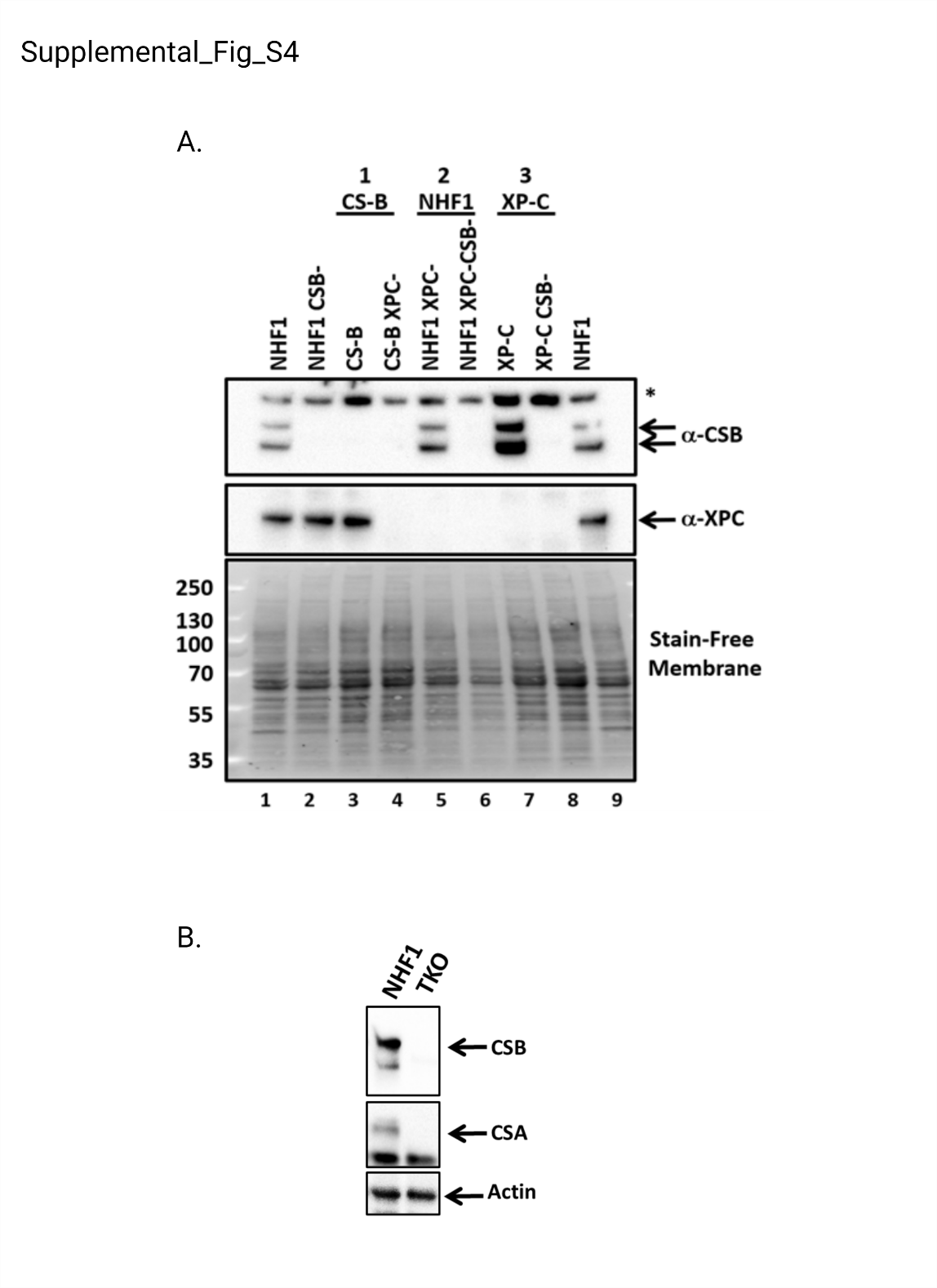
**

**Figure S4.** Western blot analysis confirms the lack of XPC, CSB, and CSA proteins in the CRISPR-Cas9 edited mutant cell lines. Proteins were extracted with Radioimmunoprecipitation assay buffer [RIPA: 20 mM Tris-HCl (pH 7.5), 150 mM NaCl, 1 mM Na2EDTA, 1 mM EGTA, 1% NP-40, 1% sodium deoxycholate] with added cOmplete™ protease inhibitor cocktail (Millipore #11836170001). Protein concentrations were determined by Bradford analysis (BioRad #5000006) and equal amounts were loaded onto 4-20% TGX Stain-Free™ protein gels (BioRad #4568095) **(A)** The three sets of parental and double knockouts are indicated at the top, and the asterisk (*) indicates a nonspecific band recognized by the CSB antibody. Fluorescent detection of total protein on the membrane is shown at the bottom as a loading control. **(B)** Wild type NHF1 and NHF1/XPC^-/-^/CSB^-/-^/CSA^-/-^ triple knock out (TKO) cell lysates were blotted for CSB, CSA, and Actin as a loading control.

**
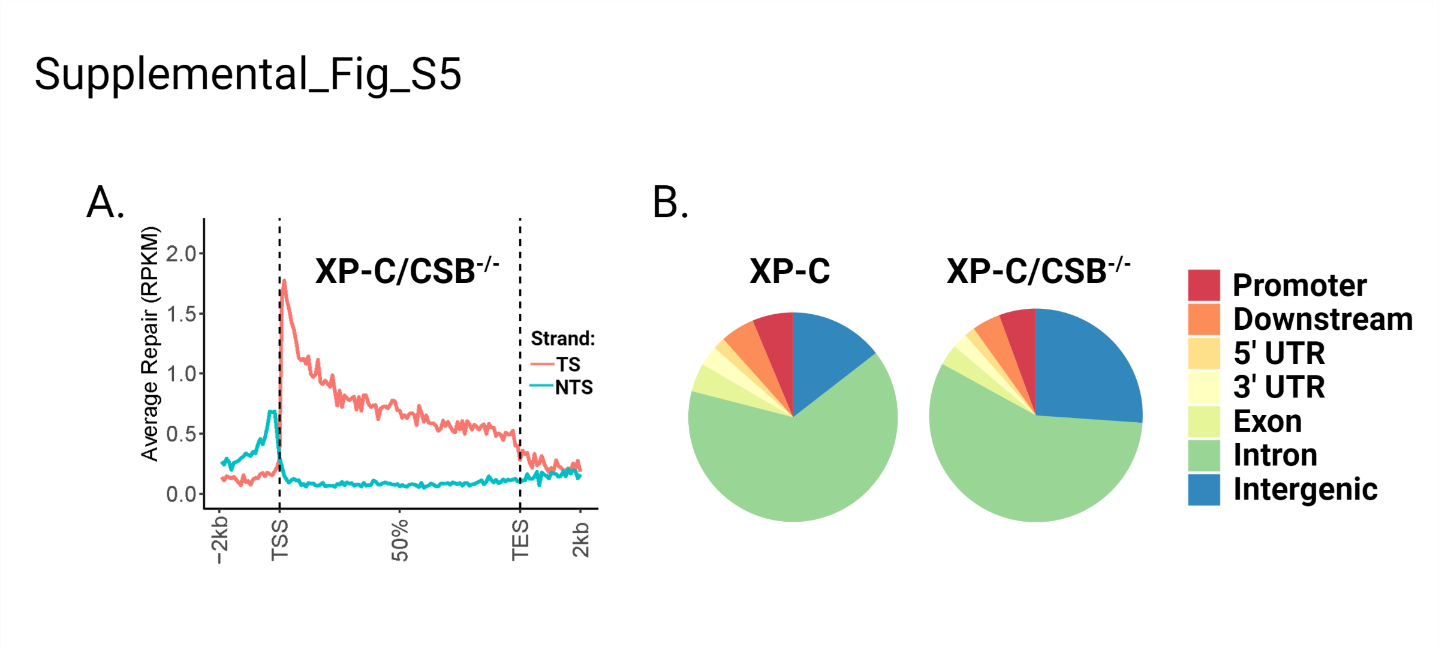
**

**Figure S5.** Transcription-coupled repair of (6-4)PP in XP-C/CSB^-/-^ cells. **(A)** XR-seq data is plotted as in Figure 1: average repair reads (y-axis) along the length of a “unit gene” (x-axis) (divided into 100 bins; 10,100 genes were selected for length > 5 kbp and no overlaps with a distance of at least 5 kbp between genes). There was low read number with CPD-containing oligos so the unbound material was used for IP with (6-4)PP in this experiment. RPKM, reads per kilobase per million mapped reads; TSS, transcription start site; TES, transcription end site. TS, transcribed strand; NTS, nontranscribed strand. **(B)** Repair as a function of genomic location. The parental XP-C cell line, which exhibits only transcription repair, has only a small proportion of repair events in intergenic regions, whereas in the XP-C/CSB^-/-^ cells, which clearly exhibit TCR in (A) have repair reads mapping to intergenic regions suggesting that there is also some global repair in these cells.

**
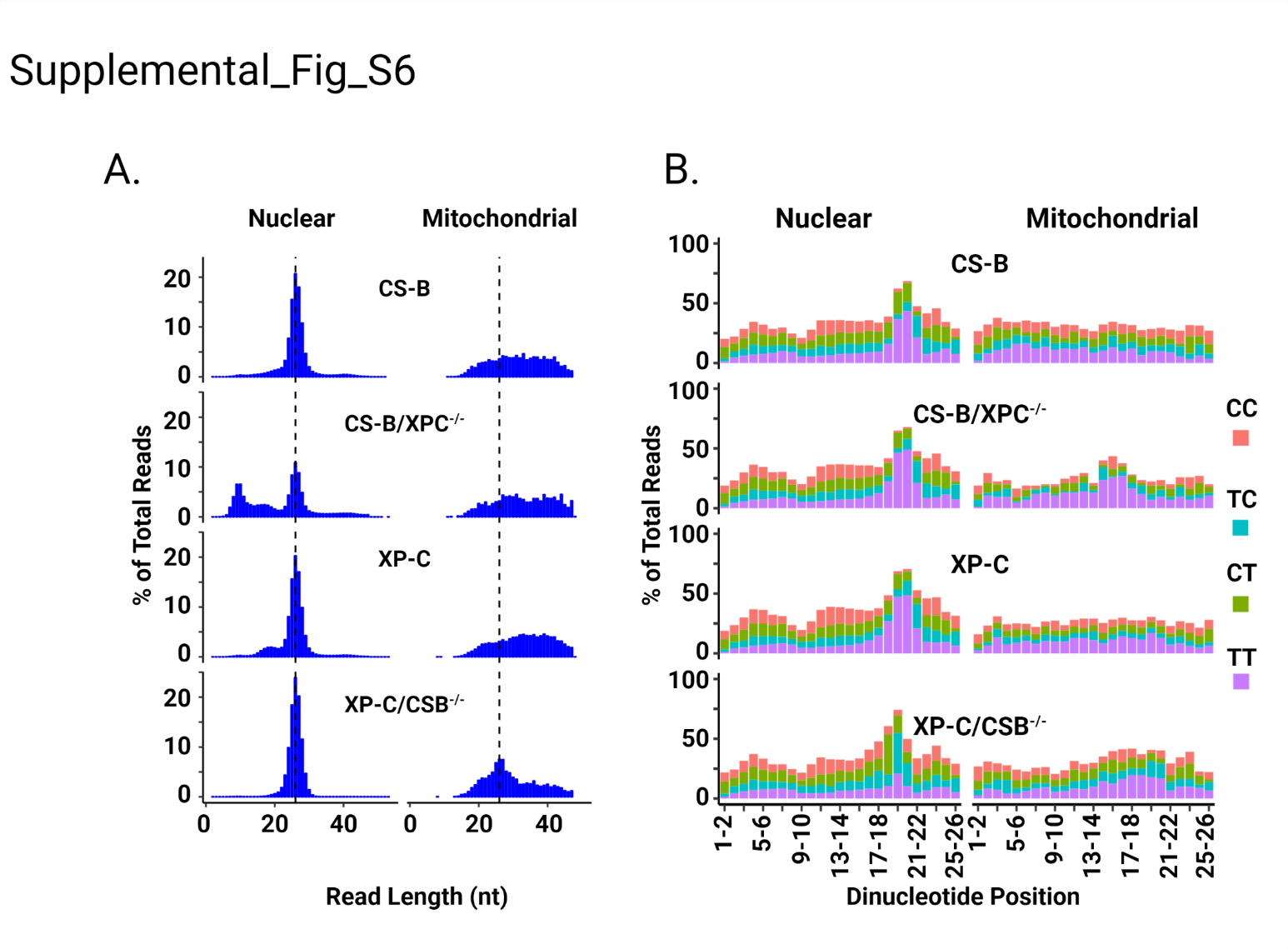
**

**Figure S6.** Patient XPC^-/-^/CSB^-/-^ cell lines excise UV photoproducts by the same dual incision mechanism as wild type cells. **(A)** Length distribution of XR-seq reads from the indicated cell lines mapped to either nuclear DNA (left) or mitochondrial DNA (right). The 26-nt median is indicated with a dashed line. **(B)** Analysis of the frequency of each of possible dipyrimidine along XR-seq reads of 26-nt length mapped as in (A). Note that the XP-C/CSB^-/-^ data set is with excised (6-4)PP oligos and thus the difference in dipyrimidine distribution. Mitochondrial DNA analysis is included to control for specificity.

**
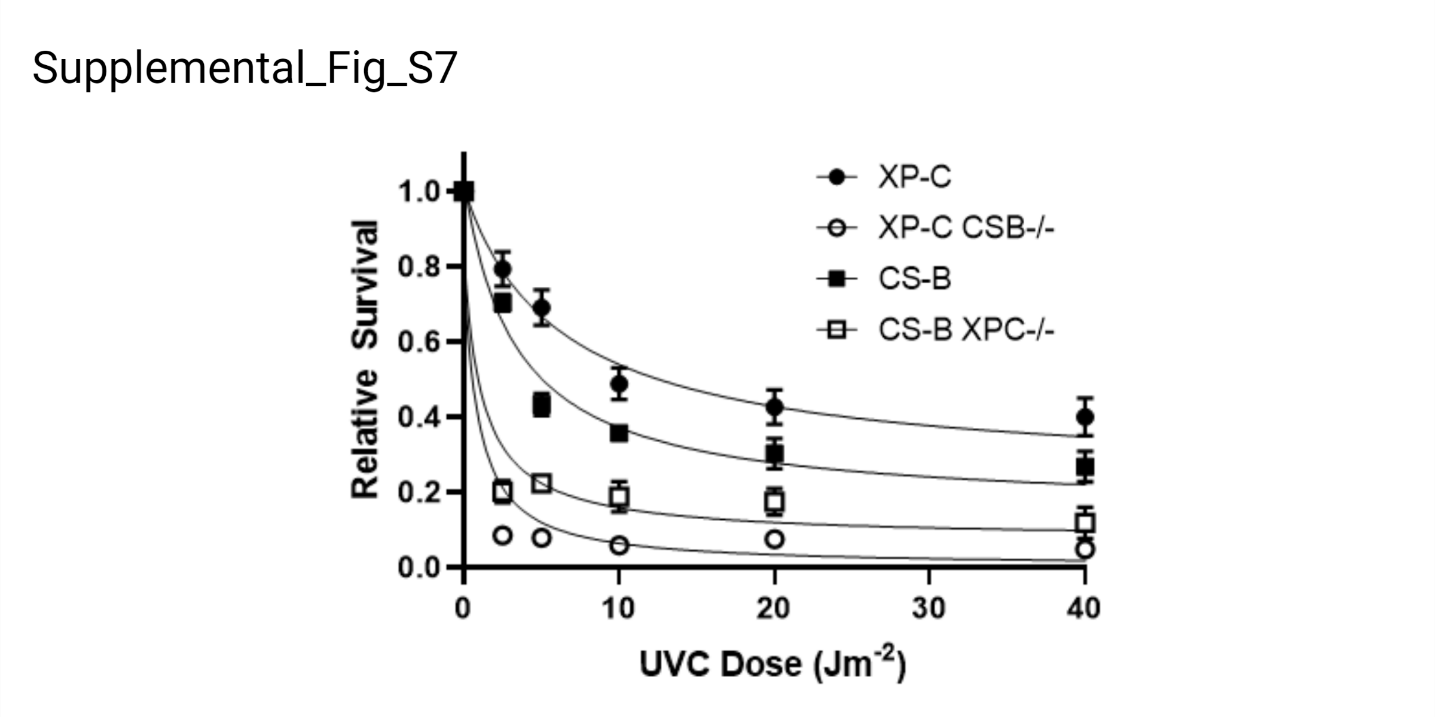
**

**Figure S7.** Patient XPC^-/-^/CSB^-/-^ cell lines are extremely UV sensitive. The MTT survival assay was performed two days after the indicated doses of UVC. Shown are the mean from three biological replicates with error bars denoting standard deviation.

**Figure S8.** XPC^-/-^/CSB^-/-^ patient and NHF1 cell lines have very low levels of (6-4)PP repair. **(A)** Slot blot analysis showing (6-4)PP repair rates of the four NHF1 cell lines treated with 5 J/m^2^ UVC. (6-4)PP signals were normalized to time=0 and plotted as a function of time. Results shown are the mean from three biological replicates with error bars denoting SD. **(B)** The Excision Assay was used to compare the amount of (6-4)PP-containing excised oligos in extracts from three the parental and XPC^-/-^/CSB^-/-^ cell lines (double knockouts in lanes 5, 7, 9 with the parental strain indicated above). An equal number of cells were irradiated with 20 J/m^2^ UVC and incubated 2h at 37°C to allow repair. Cells were lysed by the Hirt procedure and low molecular weight DNA in the supernatant was immunoprecipitated with anti-(6-4)PP antibodies. The recovered oligos were mixed with a 50-mer internal control oligo, 3’-end labeled, and separated on a DNA sequencing gel along with the indicated size markers. **(C)** Quantitation of three biological replicates of the Excision Assays above showing the mean with error bars denoting standard error of the mean.

**
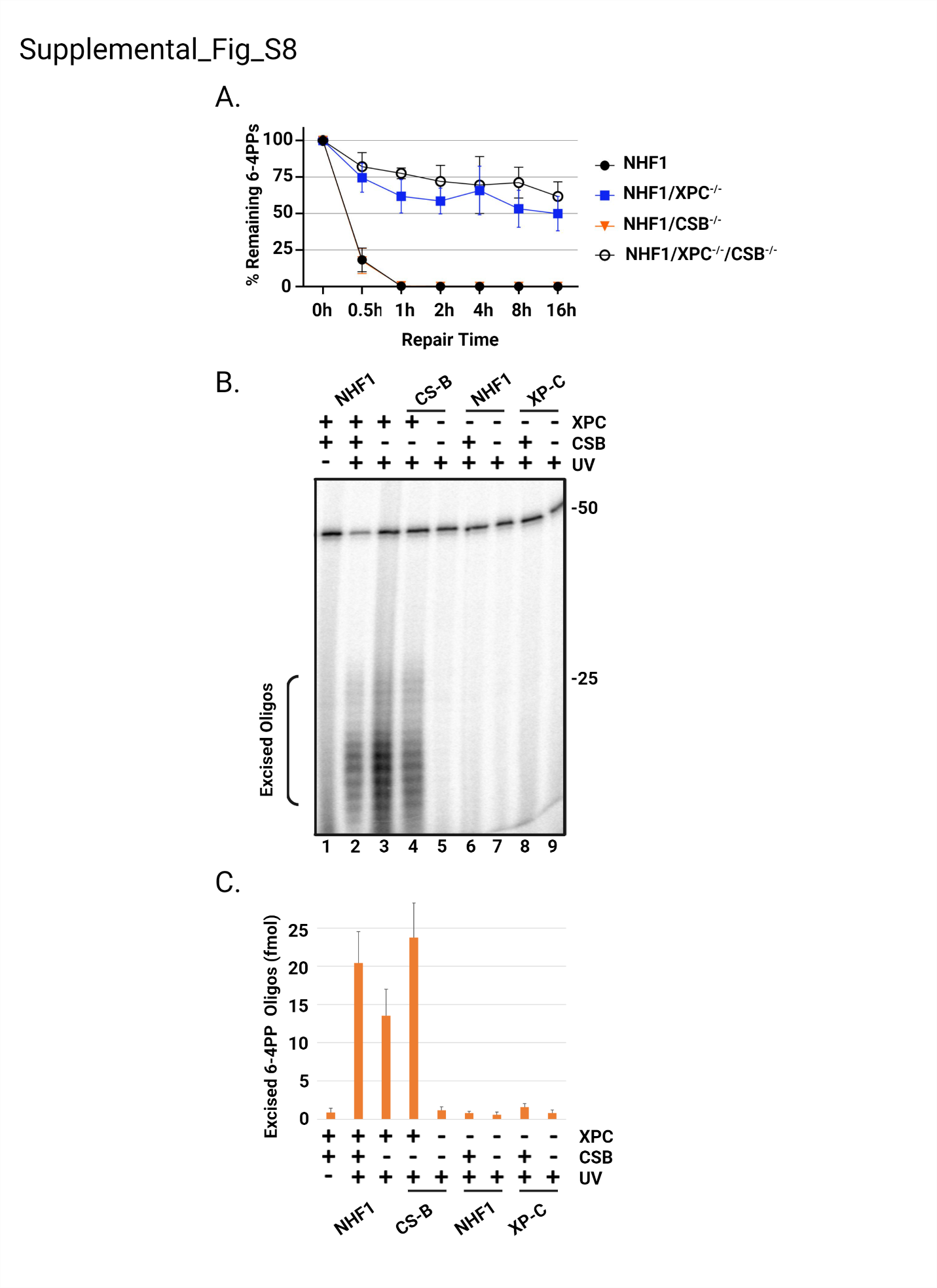
**

**
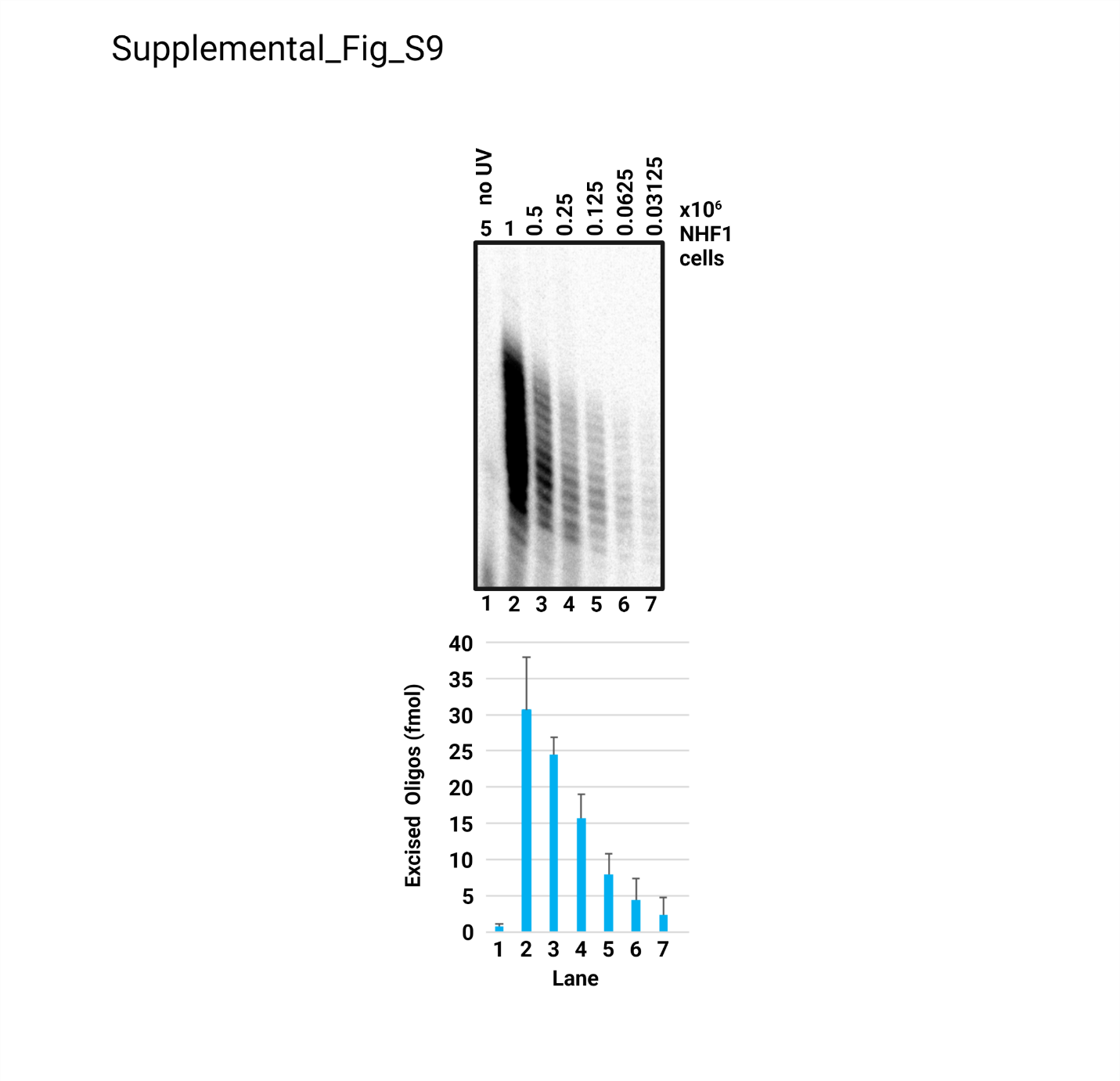
**

**Figure S9.** The Excision Assay was performed on 2-fold serially diluted NHF1 cells to determine the lower limit of the assay in order to estimate the relative amount of oligos isolated from XPC^-/-^/CSB^-/-^ cell lines. The indicated number of NHF1 cells were irradiated with 20 J/m^2^ UVC and incubated 2h at 37°C to allow repair. Cells were lysed by the Hirt procedure and low molecular weight DNA in the supernatant was immunoprecipitated with anti-(6-4)PP antibodies. The recovered oligos were 3’-end labeled and separated on a DNA sequencing gel. Quantification of two biological replicates showing the mean with standard deviation.

**
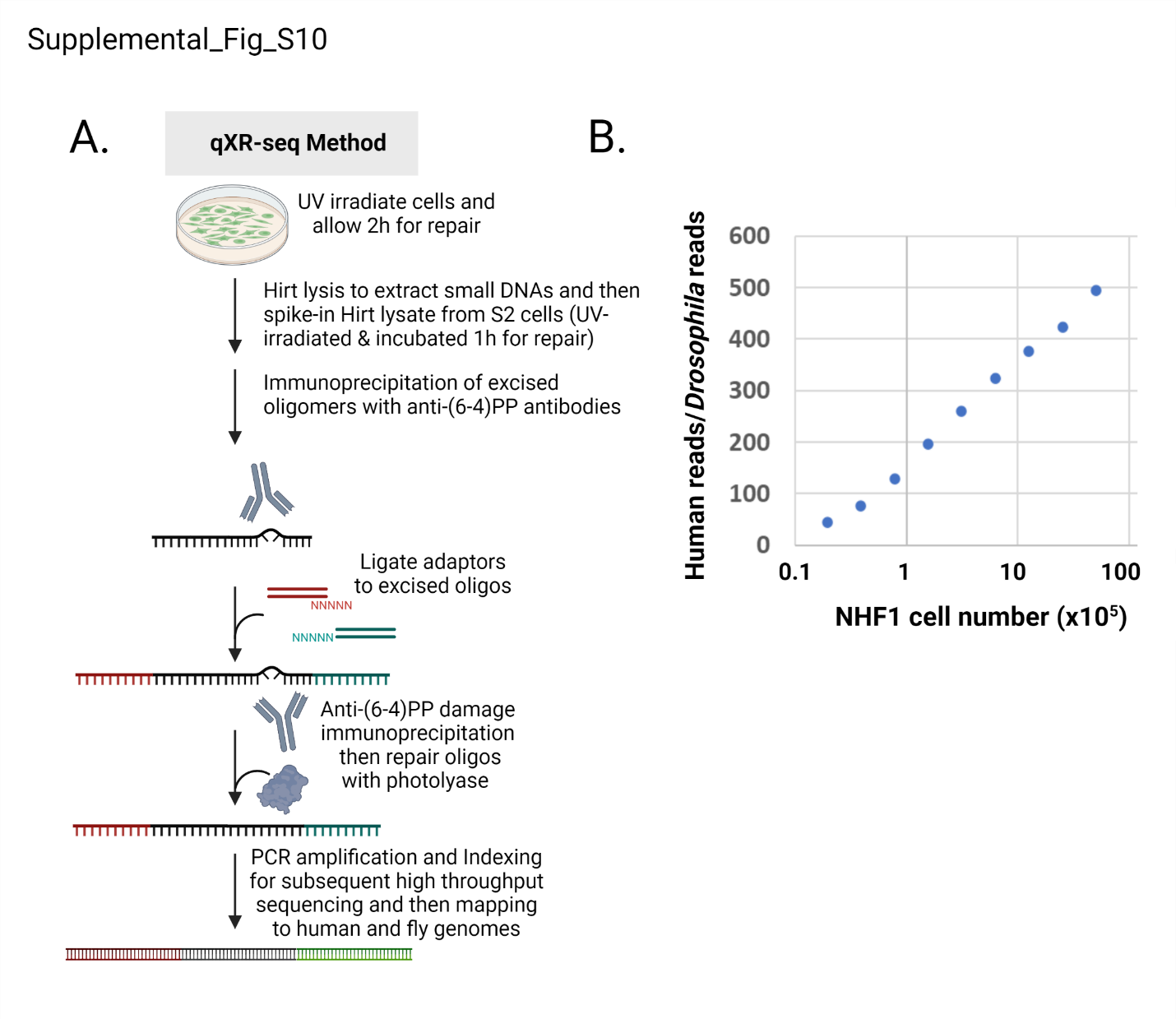
**

**Figure S10. The quantitative Excision Repair-sequencing (qXR-seq) Method. (A)** The qXR-seq method was used in Figure 5 to map the genomic location of excision products in a quantitative manner. Excised oligos were isolated from Hirt lysed human cells, mixed with excised oligos from Hirt lysed *Drosophila* cells, then purified with anti-(6-4)PP specific antibodies, ligated to adapters, and again purified with anti-(6-4)PP antibodies. The damage was reversed with 6-4 photolyase and PCR was performed to generate libraries for high throughput sequencing. **(B)** Analysis of an qXR-seq experiment in which NHF1 cells were serially diluted 2-fold before Hirt lysis and the subsequent spike-in of an equal amount of S2 cell *Drosophila* Hirt lysate before the immunoprecipitation and ligation steps. The sequencing reads were uniquely mapped to either the human or fly genomes, and since the human:fly ratio correlates linearly with the initial cell number, it can be used to quantify relative oligo amounts between samples.
